# Multiple Casual Sex Scripts: Shared Beliefs about Behavior among Portuguese Emerging Adults

**DOI:** 10.1080/19317611.2023.2172512

**Published:** 2023-02-02

**Authors:** Rita Luz, Ana Pegado, Cristina Godinho, Cícero Pereira, Maria-João Alvarez

**Affiliations:** a Faculdade de Psicologia, CICPSI, Universidade de Lisboa, Lisboa, Portugal; b Católica Research Centre for Psychological, Family and Social Wellbeing, Universidade Católica Portuguesa, Lisboa, Portugal; c Instituto de Ciências Sociais, Universidade de Lisboa, Lisboa, Portugal

**Keywords:** Casual sexual relationships, emerging adults, casual sexual scripts, sexual behavior, condom use

## Abstract

**Objective:**

Empirical research to differentiate casual sex scripts is still limited. We aimed to ascertain the sexual scripts for three main types of casual sexual relationships: *hookup*, *friends with benefits* and *one-night stands*.

**Methods:**

Through a mixed-method approach, we performed a study consisting in two sequential tasks to (1) complete three lists of script actions, and (2) identify the most agreed-upon actions for each casual sexual relationship.

**Results:**

An important number of actions and events were identified for the three casual sexual scripts, reflecting a high level of elaboration and structure. Following a cognitive-script methodology, the actions retained for the content of the script for each casual sexual relationship were those obtaining at least 60% in respect to the mean of their centrality to the encounter. Only 16.5% of actions were shared among the three scripts, demonstrating their distinctiveness.

**Conclusion:**

Knowledge about the different casual sex scripts can be used to develop relational and personal skills within CSRs and decrease unwanted experiences such as condomless sex.

A central goal of script theory is to understand how events are learned, represented, and used (Schank & Abelson, 1977). As is the case with other social behaviors, people also develop scripts for sexual interactions (Gagnon, 1990). Sexual scripts are mental representations that operate on cultural, interpersonal, and intrapersonal levels to guide expectations about sexual behavior (Gagnon, 1990; Simon & Gagnon, 1986), allowing anticipation of the sequence of predictable behaviors. If a script is available, there is a high probability that individuals will rely on it for the accomplishment of the activity, since it reduces the required cognitive effort during performance (Langer, 1978).

Changes in relational practices, detected mainly in college students (e.g., Glenn & Marquardt, 2001; Paul et al., 2000), have highlighted the relevance that casual sexual relationships (CSRs) have acquired in the lives of young adults, bringing an increase in the study of their scripts (e.g., Eaton et al., 2016; Epstein et al., 2009; Holman & Sillars, 2012). Research on sexual scripts is comprehensive and provides a framework rich in subtleties and storylines that should guide us as to the collection and analysis work to be done, whether using cognitive scripts or qualitative methodologies. Despite these inspiring works, there is a troubling gap in the study of scripts regarding CSRs, which stems from these relationships being treated as uniform interactions (for an exception see Epstein et al., 2009), without keeping up with evidence of the existence of shared knowledge of a diversity of increasingly complex casual relationships (e.g., Rodrigue et al., 2015; Wentland & Reissing, 2011, 2014) that have characteristics distinct from each other (Alvarez, Pereira, et al., 2021; Jonason, 2013).

In view of this knowledge, we anticipated different inferences and expectations for the main CSRs; we set out to study their sexual scripts with an eye for the implication these may have for a healthier experience of CSRs and sexual health in terms of preventing sexually transmitted infections through condom use, as well as of promoting a positive and respectful attitude toward sexuality, sexual activity, and sexual expression (WHO, 2017).

## Casual sex

Casual sexual relationships are usually described as non-committed sexual relationships, with one or more people, devoid of expectations of romantic attachment, regardless of how well participants know each other or of the duration of the relationship, involving a range of sexual activities from kissing to intercourse (Claxton & van Dulmen, 2013; Grello et al., 2006; Hatfield et al., 2012).

Several studies have examined the consequences of the involvement in CSR, especially on mental health, showing effects that were detrimental (e.g., Bersamin et al., 2014, Weitbrecht & Whitton, 2020), mixed (e.g., Owen et al., 2011), and positive (e.g., Shepardson et al., 2016; Vrangalova, 2015; Woerner & Abbey, 2017). These conflicting findings may, however, reflect the diversity of CSRs, as different types of CSRs may present features or occur in certain ways that permit sexual experiences that are more gratifying and safe or less so, helping to explain the variability of outcomes associated with these relationships.

The greater importance of CSRs in the life of young adults found in different sociocultural contexts (Alvarez et al., 2019; Correa et al., 2017; Wade, 2017; Wentland & Reissing, 2014) is partially outlined in the emerging adulthood theory (Arnett, 2015), which explained involvement in CSRs as a means to explore sexual identity in this phase of life, being accompanied by more complexity, which is reflected in the diversity of these experiences (e.g., Claxton & van Dulmen, 2013; Garcia et al., 2012; Wentland & Reissing, 2011). The main CSRs range from a single sexual encounter with a stranger (*one night stand*; Regan & Dreyer, 1999), to a continuous but impersonal, utilitarian relation (*booty call*; Jonason et al., 2009), to a relation that is more personal but focused on sex (*fuck buddies*; Weaver et al., 2011; Wentland & Reissing, 2011), to a relationship that is more personal and not exclusively focused on sex (*friends with benefits*; Afifi & Faulkner, 2000), borrowing the descriptions from Wentland and Reissing (2014). Additional subtly-distinguished main forms of CRSs have also been identified (Rodrigue et al., 2015), not to mention the dozen of more idiosyncratic designations, and probably experiences, found in the studies (e.g., Alvarez, Pereira, et al., 2021; Nelson et al., 2011; Singer et al., 2006; Wentland & Reissing, 2011).

The different purposes of these relationships are accompanied by several equally distinct characteristics (Alvarez, Pereira, et al., 2021; Alvarez et al., 2019; Jonason, 2013; Mongeau et al., 2013; Rodrigue et al., 2015; Wentland & Reissing, 2011) pointing away from a uniform reality toward less-expected experiences within the scope of non-committed sexual relationships, diverging from traditional conceptions, which help to validate a more nuanced perspective of the CSRs among emerging adults.

## Sexual scripts for casual sexual relationships

For many years, sexual scripts have been investigated for regular and for casual relationships, implying the presumption that the dynamics of these partnerships were distinct and mutually exclusive. More specifically, the study of sexual scripts was carried out mainly through the inspection of the sequence of the sexual events expected for first dates (Klinkenberg & Rose, 1994; Laner & Ventrone, 1998; Rose & Frieze, 1989, 1993), for romantic relationships (Ortiz-Torres et al., 2003), and for casual sex (Edgar & Fitzpatrick, 1993; Maticka-Tyndale & Herold, 1997) or, using a more contemporary term, “hookups” (Paul et al., 2000), with casual sexual relationships being conceived as uniform categories with similar purposes and interactions. In this way, comparisons between scripts were carried out mostly between the romantic script and the casual script (e.g., Eaton & Rose, 2012) and only more recently between different casual sexual relationships (Epstein et al., 2009; Holman & Sillars, 2012).

Although there are few studies examining CSRs as multiple realities and their respective scripts, some have shown that the scripts are not the same for the different CSRs, and the accumulating evidence of their distinctiveness (e.g., Wesche et al., 2018) makes it pertinent to study their scripts differently, not only for the expectations and inferences that will be generated, but also for the orientations they provide for the behavior itself. Several authors have highlighted the completeness of mixed-methods in the study of scripts, which make it possible to provide both greater depth and breadth of information (e.g., Bowleg et al., 2015; Sakaluk et al., 2014).

## Casual sex scripts and sexual health

Risky sexual scripts are significantly correlated with risky sexual behaviors (Bowleg et al., 2015; Tomaszewska & Krahé, 2018) because sexual scripts make the actions that are part of them mentally accessible and, therefore, more influential in guiding behavior. One of the reasons why knowing whether the condom is part of the script can be so relevant in terms of sexual health is that in situations in which behavior is guided by the script, the presence of condoms in these cognitive representations increases the likelihood that they will be used in sexual interaction, as supported by research (Alvarez & Garcia-Marques, 2008; Maticka-Tyndale & Herold, 1999).

It is well known that condoms are used more in casual relationships than in regular ones due to the increase in the perception of security and trust that results, among other aspects, from the feeling of familiarity developed (e.g., Misovich et al., 1997). However, Bowleg et al. (2021) drew attention to the possibility that condom use might differ by variations within casual partnership type and dynamics, the same having already been found in sex-only partnerships, where women frequently used condoms, but their use became less common as relationships continued, even if the partnerships remained casual (Lima et al., 2018). These results are in line with studies where casual sexual scripts (Lenton & Bryan, 2005) or hooking up scripts (Holman & Sillars, 2012) were ascertained, as no reference to condom use was found in these scripts, even when sexual intercourse occurred. Condom absence has also been found in previous studies on the description of the typical script for hookups (Alvarez & Garcia-Marques, 2008; Downing-Matibag & Geisinger, 2009; Paul & Hayes, 2002). Hence, we anticipate that condom use will not be part of all CSR scripts, that it will be present in the *one-night stand* script, and that with increasing level of partner familiarity, condom use will be absent and thus in great need of targeting for promotion.

Besides the avoidance of risky sexual behaviors such as condom less casual sex, sexual health also refers to an overall state of well-being regarding sexuality, as well as a positive approach to sexual relationships (WHO, 2017). Sexual pleasure, which is a core motivation for engaging in casual sex (Luz et al., 2022), is defined as the “physical and or psychological satisfaction and enjoyment one derives from any erotic interaction” (Philpott et al., 2006) and constitutes an important aspect of sexual health, being considered essential for overall health and wellbeing (Coleman et al., 2021). Further knowledge about casual sex scripts may provide essential information concerning elements and actions that may contribute to sexual pleasure and sexual health.

## The present study

Sexual scripts are culture-specific guides, and their investigation in other cultural contexts is very relevant given the possibility of populations having different cultural norms and rules for getting together sexually.

We propose to investigate the scripts of different CSRs, and the place of condoms in them, in the same study using a mixed-methods methodology, first qualitative and then quantitative, and to do it in a culture outside the US, where the study of CSRs has yet to be carried out. As in most of the literature on sexual scripts, we focus on interpersonal scripts that rest on cultural scripts to guide individuals through the particulars of each sexual encounter, a complex act involving mutual dependence (Simon & Gagnon, 1986). Instead of studying these scripts limited to samples of college students, we also included non-college participants and those of any sexual orientation, as has been recommended in the literature (Williams & Harper, 2014).

## Method

### Overview

We used a mixed-method approach, performing two sequential tasks (*N* = 149), to establish the sexual scripts for the main CSRs known of or experienced by Portuguese emerging adults. The goal of the first task was to complete the lists of script elements (actions and events) by adding actions participants considered to be usually present in the CSRs. The list of actions and events were obtained in a previous qualitative study (Alvarez, Pegado, et al., 2021; Luz et al., 2022), from which stemmed, among other themes, an important body of information concerning the beginning and ending of each CSR, sexual experience and sexual protection, the role of new technologies, and underlying scripts and rules. The need to complete these lists arose from the fact that during those focus group interviews there was no explicit request or instruction to describe what occurred from the beginning to the end in each CSR, and all actions were spontaneously reported by the participants (Eaton & Rose, 2012).

In the second task we aimed to identify the most central script elements for each CSR following a cognitive-script methodology, according to which script content is determined by high agreement in the actions mentioned by more than 25% of participants in free-recall tasks (Bower et al., 1979). A more stringent criterion of 50% has been advocated for the checklist format due to its possibility of having a high level of detail and a greater frequency of various actions (Eaton et al., 2016; Eaton & Rose, 2012). The suitability of the sequence of the script elements was evaluated.

For both tasks, inclusion criteria for participating were: being aged between 18 and 29 years, speaking European Portuguese as one’s native language, and having had at least one sexual relationship (oral, vaginal, or anal). Ethical approval was granted by the FPUL Ethics Committee (n.1 2017_18), and all participants signed an informed consent form, completed a sociodemographic data questionnaire (see Table 1), and were compensated by receiving a 5€voucher for their participation.

**Table 1.** Demographic Characteristics of the Samples.

	Study 1 (*N* = 61)	Study 2 (*N* = 88)
	*M* ± *SD* (max, min)	*M* ± *SD* (max, min)
Age of first intercourse	17.02 ± 1.9 (13, 21)	17.18 ± 2.1 (13, 26)
Age of first CSR	18.11 ± 2.3 (14, 22)	18.54 ± 3.0 (13, 28)
Gender	*N* (%)	*N* (%)
Women	47 (77)	49 (56.3)
Men	14 (23)	38 (43.7)
Religion		
Non-practicing	40 (65.6)	62 (71.3.6)
Catholic	19 (31.1)	23 (26.1)
Other	2 (3.2)	2 (2.2)
Occupation		
Student	48 (78.7)	41 (47.1)
Employed	6 (9.8)	26 (41.4)
Working student	7 (11.5)	5 (5.7)
No occupation	0 (0)	5 (5.7)
College year		
Undergraduates	11 (23.9)	16 (39.0)
High-school	1 (2.2)	1 (2.4)
1st year	3 (6.5)	1 (2.4)
2nd year	2 (4.3)	5 (12.2)
3rd year	4 (8.7)	9 (22.0)
4th year	1 (2.2)	–
Post-graduates	35 (76.1)	25 (61.0)
Sexual partner gender		
Only men	41 (69.5)	43 (48.9)
Mainly men	2 (3.4)	7 (8.0)
Both men and women	0 (0)	3 (3.4)
Mainly women	3 (5.1)	1 (1.1)
Only women	13 (22.0)	34 (38.6)
Sexual orientation		
Heterosexual	54 (90.0)	75 (85.2)
Bisexual	3 (5.0)	8 (9.1)
Homosexual	2 (3.3)	4 (4.5)
Pansexual	1 (1.7)	1 (1.1)
Relationship status		
Single	27 (44.3)	36 (41.9)
Dating	32 (52.5)	49 (57)
Unmarried couple	0 (0)	1 (1.2)
Married	1 (1.6)	–
Divorced	1 (1.6)	–
CSR experience		
Yes	42 (68.9)	57 (64.8)
No	19 (31.1)	31 (35.2)
Currently	(*N* = 42)	(*N* = 57)
Not involved in CSRs	35 (83.3)	44 (77.2)
Involved in one CSR	7 (16.7)	10 (17.5)
Involved in several CSRs	0 (0)	3 (5.3)

#### Task 1 – Completing script elements

##### Participants

The sample included 61 participants, of ages between 18 and 29 years old (*M* = 22.72; *SD* = 2.7), 77% of whom were women (see Table 1). The lifetime number of sexual partners ranged from 1 to 8, with a modal value of 3 partners. Concerning the lifetime number of casual sexual relationships, this varied from 1 to 8, and the mode was 1.

##### Materials and procedure

For the development of the initial lists of script elements, one author (RL) reread and collected all content concerning the beginning and ending of each CSR, sexual experience and sexual protection, the role of new technologies, and underlying scripts and rules, specifying to which CSR each excerpt referred (*one-night stand*, *friends with benefits*, *hookup*). Afterwards, two authors (MJA and RL) reread all excerpts and independently selected short sentences that contained script elements for each CSR. The level of agreement concerning the script elements to be included was calculated (Cohen’s K between .70 and .80), and minor disagreements were solved in discussion between both researchers.

Prior research investigating casual sexual scripts was also consulted in order to better formulate action items and bring them together in a logical sequence of events and actions. Therefore, following Eaton and Rose (2012), an action was defined as a verb, i.e., a word or words that in syntax conveys an action (e.g., dance, drive, talk), an occurrence (e.g., happened, became), or a state of being (e.g., be, feel). In parallel, inspired by literature on the phases of the sexual interaction and on the main scenarios found in casual sexual encounters (e.g., Bowleg et al., 2015; Landgraf et al., 2018; Lenton & Bryan, 2005; Olmstead et al., 2019), the authors created a framework to accommodate the participants’ knowledge and arrange the more specific events and actions in the sexual script of each CSR. This initial framework contained six moments in the sexual encounter, each one divided into several categories.

The two researchers subsequently allocated all script elements identified for each CSR to one moment and one category. After this procedure, some modifications had to be made to the framework; in its final version it contained four moments and eighteen different categories, with some variations among the three CSRs: context (time and place, emotional involvement, and partner types); approach (technology use, social network, planning, flirting, verbal communication, motivations, and defining rules); sexual experience (sexual initiation, sexual intercourse, and condom use and communication); and post-intercourse (post-intercourse communication, post-intercourse technology use, outcome with partner, continuity and duration, and termination). This resulted in three lists containing 68 script elements organized in 15 categories for *one-night stand*; 44 script elements organized in 15 categories for *friends with benefits*; and 30 script elements organized in 11 categories for *hookup* (Supplementary file).

Material with the three lists of script elements to complete (*one-night stand*, *friends with benefits*, *hookup*) was developed using an online platform (Qualtrics). The material included the definition of each CSR (Appendix) and the following instruction: “*Despite the variability of what happens in this relationship, there are some elements that are more typical or usual to occur and that we are interested in knowing in a more complete way. In the following pages you will find a list of events and actions that were considered frequent during this relationship. We ask you to write down any missing actions that you think complete the sequence of events belonging to this specific relationship. One line for each action/event. Do not describe actions or events that you may have experienced but rather those that generally show how such an encounter could take place between two individuals*.” A pilot study with sixteen participants was performed in order to ensure that instructions were clear.

Eighty-six participants followed the anonymous link to the questionnaire, signed the informed consent, and accessed the material. A random distribution of the three lists was programmed so that each participant completed the list of script elements concerning only one CSR.

From these, 22 participants (18 women) completed the *one-night stand* questionnaire, 23 participants (18 women) completed the *friends with benefits* questionnaire, and 16 participants (11 women) completed the *hookup* questionnaire, resulting in 61 valid questionnaires. Eighteen questionnaires (eight women) were considered null and excluded from data analysis because they were incomplete and 7 participants (three women) reported in the sociodemographic data form never having had oral, vaginal, or anal sex and therefore were not included in the study.

##### Data analysis

Initially, in order to attain methodological accuracy, both researchers randomly selected and read the set of new action items added by participants for two of the forty-one categories (15 categories for *one-night stand*, 15 for *friends with benefits* and 11 for *hookup*). In each category, the authors performed an independent qualitative analysis by aggregating action items considered similar or equivalent into a single script element and excluding those analogous to the ones previously presented to participants. Moderate to strong levels of agreement (Cohen’s K between .74 and .87) were obtained between both categorizations and divergences were resolved by both authors. To pursue the analysis, 12 (30%) of the remaining thirty-nine categories were randomly selected and analyzed. Moderate levels of agreement between both authors were obtained (Cohen’s K = .72) and mismatches were resolved through discussion. Considering the levels of agreement obtained, the same procedure was applied to the remaining twenty-seven categories, and, in order to overcome idiosyncrasies in the characterization of the CSRs, only action items that were mentioned by three or more participants were selected as potential script elements.

##### Results and discussion

Participants added a total of 1,170 new script elements to the three lists: 457 to the *one- night stand* list, 464 to the *friends with benefits* list, and 249 to the *hookup* list. After qualitative data analysis, 30 new script elements mentioned by three or more participants were added to the *one-night stand*, 35 to the *friends with benefits,* and 27 to the *hookup*, resulting in three completed lists of script elements: *one-night stand* with 98 script elements, *friends with benefits* with 79 script elements, and *hookup* with 57 script elements (Supplementary file).

The number of elements of a script is an indicator of the richness and structure of that script (Eaton & Rose, 2012). The considerable number of initial script elements together with the new script elements shows that the CSRs are well-known and that Portuguese young adults are familiarized with the actions and events that should occur during each CSR. New script elements added by participants in this study conferred more structure and detail to casual sexual scripts, confirming the benefit of completing the list of actions previously generated spontaneously (Eaton & Rose, 2012). Contrary to the idea that, unlike committed relationships such as dating, the scripts for CSRs may be less accurate (Bradshaw et al., 2010; Eaton & Rose, 2012), this study provides evidence that CSRs have existed long enough so that emerging adults have been able to develop thorough, coherent, and consensual casual sexual scripts.

The *one-night stand* script is the one presenting the most script elements. It seems to be the most prototypical CSR, implying that participants report more actions and events that frequently occur in these encounters as they are more easily accessible at the cognitive level when the script is enacted (e.g., Eaton & Rose, 2012; Olmstead et al., 2019). *Friends with benefits* also presents a well-structured script, with an important number of script elements, indicating that this CSR is also relevant for the sample and that, contrary to other findings that suggest it being more an ideal than a real occurrence (Epstein et al., 2009), this relational arrangement is frequent and viable. The *hookup* script included fewer script elements. One possible explanation is that, because the material for this script was presented to a lower number of participants, fewer actions and events were added to this list. However, as found by Alvarez, Pereira, et al. (2021), this CSR is less associated to an agreed-upon label and definition than *one-night stand* and *friends with benefits*, probably because it includes more idiosyncratic and personal experiences and hence a wider array of possible actions and events during the interactions. Consequently, we also found a lower social consensus about the script elements making up this script, resulting in the inclusion of less actions and events.

Because this was a free-recall task, participants may have provided more subjective and idiosyncratic script elements, based on their own personal experiences. Even though only action items that were mentioned by three or more participants were selected as potential script elements, it seemed important to identify the actions and events that are more consensually considered present (Bower et al., 1979) in each CSR. We hence performed Task 2, using a checklist format through which participants could indicate the frequency of each script element, in order to ascertain the script content for each CSR.

#### Task 2 – Consensual script elements

##### Participants

The sample comprised 88 participants, of ages between 19 and 29 years old (*M* = 23.75; *SD* = 2.9), 56.3% of whom were women (see Table 1). The participants had had between 1 and 15 sexual partners in their lives so far, with the modal value being 1 partner. Concerning the number of CSRs indicated by participants who said they had already had casual sexual relationships, this varied between 1 and 20 throughout life, and the mode was 1 to 2 CSRs. The evaluation of the sequence of script elements was made by a sample of 15 participants (*M_age_* = 25, *SD* = 2.90), 53.3% of whom were women.

##### Materials and procedure

We developed an online questionnaire (Qualtrics) presenting the definition of the presented CSR (Appendix) and the following instruction: “*Indicate how often (%) each event or action is present/occurs in this casual relationship. For instance, if you think that ’there is physical attraction’ is present in 50% of the sexual encounters in this relationship, you must indicate this value on the presented scale and continue to evaluate the next events or actions. Thinking about this CSR, indicate the percentage value that you consider to best represent how often each action or event occurs in this CSR. Do not attribute higher frequency to actions or events that you have experienced yourself, but rather those you consider to usually happen in this type of relationship.*” After the instruction, participants were presented the lists of script elements that resulted from Study 1. We maintained the organization of script elements in categories (98 script elements in 15 categories for *one-night stand*, 79 script elements in 15 for *friends with benefits* and 57 script elements in 11 for *hookup*) and in sequence, aiming to aid participants to better contextualize each script element and thus make a more accurate estimation of how often each event or action is present in the CSR. The response scale was presented from 0 to 100, divided into 10 units. As in Study 1, a random distribution of the three lists was programmed so that each participant evaluated the list of script elements concerning only one CSR. A pilot study with five participants was performed to ensure that the instructions were clear.

We applied convenience and snowball sampling procedures by advertising the study in our social network and asking the potential participants to forward the questionnaire link to their own social contacts. One hundred and thirteen participants accessed the questionnaire. From these, 11 participants (8 women) were not included in the study due to having reported in the sociodemographic data form never having had sexual relationships, and 14 participants (9 women) were excluded due to having left their questionnaires substantially incomplete. Eighty-eight questionnaires were considered valid, and the sets of actions were evaluated by 32 participants (21 women) for *one-night stand*, 26 participants (13 women) for *friends with benefits*, and 30 participants (15 women) for *hookup*.

Two of the authors (RL and MJA), independently, used the central actions of each casual sexual script to write script-based narratives to be compared, and a final version for each casual script was reached by consensus. The suitability of the sequence of each script was confirmed by a new convenience sample who were asked if the sequence seemed adequate and if not, to make suggestions. The data was collected during in-person meetings where participants were presented with paper tags, each one containing a script action, numbered and sequentially organized. Firstly, participants were to verify and indicate whether or not they agreed with the proposed sequence of actions and events that usually occur during an encounter. Whenever participants did not agree with the sequence, a second task was proposed in which they were asked to manipulate/move the paper tags in order to organize the script elements in the sequence they thought was more adequate. A pilot study with two participants was performed in order to guarantee that the instructions were clear and well understood by participants.

##### Data analysis

The centrality of each action and event in the script was determined by calculating the mean of the values attributed by participants (between 0 and 100), reflecting whether the element was a more or less important constituent of the script. Actions and events that were considered by participants as being present in at least 60% of the encounters in each type of CSR were considered central for the script.

In order to compare the three scripts and determine to what extent they differed from each other, we organized the script elements in a logical sequence using the same four categories proposed by Landgraf et al. (2018): *approach*, representing various forms of interaction that precede physical sexual contact (e.g., encounter, talking, casual flirting, or going for a walk together); *foreplay*, consisting of physical sexual contact between sexual partners without any form of penetration (e.g., touching, cuddling, kissing); *sex and condom use*, defined by sexual relationships such as oral, vaginal, and anal penetration and the use of condoms; and *post-intercourse*, consisting of actions that occurred after sexual interaction (e.g., talking, caressing, text messaging).

For the analysis of the final sequence of actions in each script, the value of each action or event in the sequence could range from 0 to 5, with 0 indicating that all participants considered that the action should change place and 5 when everyone kept it in the same place in the sequence presented. Whenever the place where the action/event was located obtained the highest frequency, its place in the sequence was kept. The place in the sequence was changed whenever there was a majority of another action or event selected by the participants.

##### Results and discussion

A total of 109 actions or events, ranging in number between 30 in *hookup* and 44 in *one-night stand* (see Table 2), were considered by participants as being present in at least 60% of the encounters, having obtained a mean of 60% or above of the responses.

**Table 2.** Number of Actions According to Mean values of Frequency in The Script.

		Mean of values (0–100%)		
	Total	≥60%	60–69.9%	≥70%
Friends with benefits	79	35 (44%)	26	9
One-night stand	98	44 (45%)	23	21
Hookup	57	30 (53%)	16	14
Total	234	109	65	44

Scripts were qualitatively distinct as only 18 out of the 109 actions (16.5%) were common to the three scripts, 6 actions per script (happen mostly at night; individuals start by talking, even if there was mostly non-verbal communication before; there is physical attraction; no obligations; they have intercourse; and technology use before or post-sex). The script of *friends with benefits* shared 14 out of 79 actions (17.7%) with the script of *one-night stand* (in addition to those mentioned before the individuals always use a condom) and 18 out of 65 (27.7%) with the script of *hookup* (in addition to those mentioned before, individuals do not act as partners in front of most people; give kisses, touching and groping; and to finish stop sending or replying to messages). The script of *one-night stand* shared 16 out of 74 actions (21.6%) with the script of *hookup* (in addition to those mentioned before, there is no passion or emotional involvement; and individuals show interest with their gaze). The same actions sometimes appeared in different moments in different scripts; this occurred for four actions or events (there is physical attraction; there are no obligations; individuals show interest with their gaze; and the use of technology).

The qualitative analysis of the scripts was divided into four moments: approach, foreplay, sex and condom use, and post-intercourse (Supplementary file).

### Approach

The scripts are not differentiated in terms of time and place, being referred to as occurring mainly at night, although the locations for a *hookup* can vary substantially and happen in a variety of social events, such as festivals or parties, and alcohol is an important characteristic of the *one-night stand*.

The partners are mostly unknown in the scripts for *one-night stand* and *hookup*, and the feelings involved in the three scripts are different and can be characterized by a continuum of greater to lesser emotional involvement. Trust, respect, and caring are part of what is expected in *friends with benefits*; physical attraction is highlighted for the *hookup*; being emotionally detached is the affective tone of the *one-night stand*.

There is no planning beforehand in a *one-night stand*, so technology is not used to arrange the meeting. Its use and the messages exchanged are otherwise much more specific, diverse, and frequent in the preparation of a *hookup*, compared to *friends with benefits*. Consequently, the social network of friends in the organization of the outing and in the steps that precede contact with the other has a prominent role in the *one-night stand* script, absent in the other scripts.

Face-to-face flirting is the prototypical way found to create the conditions for a *one-night stand* to happen, which itself involves a mini-script within the script, with 10 sequential actions from non-verbal signs of interest such as eye contact, to approach, to a seemingly accidental physical contact, and a (minimal) verbal interaction, which is not part of the other two scripts.

Verbal communication occurs in the three scripts. In *friends with benefits* the content of the conversation stems from prior knowledge. In the *hookup* it seems above all to serve the function of increasing the ease between individuals and of finding out whether there is any type of compatibility that makes involvement possible. In the *one-night stand* verbal communication serves above all to show sexual interest and to be instrumental in the realization of sexual interaction.

There are several motivations that trigger the scripts, and the only stated motivation that was present in the three scripts was physical attraction, the motivations being more numerous and diversified in the *one-night stand*.

The defining rules (e.g., what the relationship is, its rules, and how it concludes) are part above all of the scripts of *friends with benefits* and *hookup*, especially those rules relating to behavior in social situations – not acting as partners – and the knowledge that friends and acquaintances should (not) have of it, it being possible for the *hookup* to be terminated at any time. However, in the three scripts there is a lack of obligations, explanations, and commitment.

### Foreplay

The beginning of sexual contact is present in a different way in the three scripts, with the demonstration of interest and evaluation of the other’s interest being the most important in the *one-night stand* script, probably in order to indicate the possibility of pursuing the encounter. In the *hookup*, the script includes the steps that physical contact must “obey” so as not to precipitate unwanted behaviors; in *friends with benefits* this initial sexual contact includes a range of intimate behaviors and is focused on sexual behaviors in themselves rather than on making the purpose of behaviors intelligible or their cadence appropriate.

### Sex and condom use

In all scripts sexual intercourse is expected; it is expected to be fast and intense in the *one-night stand*, but much more qualified in *friends with benefits*, being tailored by exchanges that contribute to exploration of sexuality, resulting from the complicity and feeling of being at ease sexually that is generated in these relationships. Sexual intercourse is not expected in the first *hookup* meeting.

The condom is part of the script for the *one-night stand* and *friends with benefits*, but it is unclear whether it is used. In *friends with benefits*, using a condom is quite typical, but so is the partner talking about whether they are going to use it or not. Condom use is equally prototypical in the *one-night stand*, but there is also reference to its being used with people who do not know each other.

### Post-intercourse

In the *friends with benefits* script, relaxing together and talking after sex – about whether it was good, or small talk – are expected. This post-sex verbal communication is absent in the other two scripts. The use of technology is part of the three scripts. In *friends with benefits* technology is used to arrange encounters or, by reducing these contacts, to show that one is no longer interested, or to terminate the “benefits” in the friendship; in the *one-night stand*, it is used to exchange social networks and phone numbers after the sexual encounter; and in the *hookup* it is used to signal that one is no longer interested in continuing and to terminate the *hookup*.

The development of romantic feelings on the part of one of the partners is an outcome considered typical in the *friends with benefits* relationships, not mentioned in the other two CSRs. Another aspect mentioned only in the *one-night stand* script concerns the fact that the encounters can be repeated if both enjoyed the experience. This feature is at odds with the definition of what is considered a *one-night stand*, but may result from the transformations of the CSRs and their resulting variants. For example, a *booty call*, a continuous but impersonal, utilitarian relation, may have been a *one-night stand* or *hookup* at its onset.

As far as the termination of the relation is concerned, this information is contained in the *friends with benefits* and *hookup* scripts, but absent from *one-night stand* – more in line with what would be expected, since the *one-night stand* usually does not have continuity. The script is informative about the signs that are used to show that one does not want to continue the relationship, mostly via social networks – either by taking longer or not responding at all to written messages. The script is also informative about the reasons for the withdrawal in *friends with benefits*. One of the main reasons is the development of romantic feelings, already referred to by participants as a possible outcome with the partner, with the individual who does not have these feelings being the one who usually withdraws. Another reason pertains to one of the individuals showing an interest in someone else.

### Sequence of actions and events

The given sequences for *hookup* and *friends with benefits* remained unaltered by the participants. For the *one-night stand* script, 17 changes in the order of the script were indicated and taken into account. The final versions for each script are in the Appendix.

Despite sharing some elements of the total script structure, the three casual sexual scripts are distinct from each other. The script elements that are simultaneously present in the three casual sexual scripts seem to correspond to the more prototypical aspects of CSRs, namely that they happen at night, are mostly motivated by physical attraction, involve sexual practices from kissing and groping to intercourse, imply no obligations between partners, and use technology before or after sex. Given that casual sexual scripts influence attention, memory, and behaviors (Fiske & Taylor, 1991), and guide judgments of sexual intent (Lenton & Bryan, 2005), it is of foremost importance that they be highly structured and distinguishable. This knowledge about the actions and events comprising each casual sexual script helps to reduce ambiguity and erroneous expectations, including the risk of misperception of sexual interest and the negative interactions that may arise, such as sexual coercion and sexual harassment (Haselton, 2003). This knowledge is therefore crucial in supporting CSR partners to better identify the type of sexual interactions in which they are involved, allowing them to pursue more suitable behaviors and expectations for the relationship.

Most actions and events present in the three casual sexual scripts are different, making it possible to distinguish them. This confirms that CSRs are not uniform categories, as they are not only identified with specific labels and understood as more different than similar in a set of psychoemotional, behavioral, and sexual features (Alvarez, Pereira, et al., 2021), but they are also assigned different patterns of sexual interactions that become socially accessible, helping individuals to be aware of the sequence of action and events occurring during each CSR.

### General discussion

The main contribution of the present study is to overcome the gap in sexual scripts research by exploring and comparing the scripts of different casual sexual relationships, using a mixed methodology. Deeper knowledge of the script content for different types of CSR makes it possible to identify the actions that have an impact on sexual health and wellbeing – those related to risky sexual behaviors, such as the absence of condoms in casual sex, as well as those that contribute to the promotion of positive sexuality, including sexual expression and behavior such as communication and exploration of sexuality, and so contribute to the possibility of having an enjoyable and pleasurable sexual experience.

An important number of actions and events comprise each of the three casual sexual scripts, reflecting a high level of elaboration and structure. It is known that scripts constituted by a small number of elements entail greater variability in the behaviors and rules involved, which may confer a more unpredictable quality to the encounter, thereby increasing the risk of divergent expectations about their sexual and relational outcomes (Lenton & Bryan, 2005). Identifying a higher number of actions present in each script may hence be valuable in reducing uncertainty and efficiently guiding behaviors and expectations during casual sexual interactions, with participants being attuned to the same detailed sequences of behavior during the sexual interactions (Eaton & Rose, 2012), which in turn may contribute to a more pleasurable sexual experience.

Contrary to what was found in other studies (Eaton et al., 2016), we did not find a high number of actions and events shared across the different CSR scripts. While Eaton et al. (2016) focused on the first encounter of any kind of romantic (sexual) relationships, in our studies we specified the type of relationship, which may have led to more distinct script elements. Despite this major distinction, it may not always be clear to individuals what kind of CSR they are entering, and the decision about which script to enact may only occur as the encounter evolves.

The *one-night stand* script involves actions and events associated with casual sex encounters including alcohol consumption, emotional detachment, and spontaneity of the encounter, as supported by previous research (e.g., Claxton & van Dulmen, 2013). One interesting aspect that emerges as central in this script is the role of the social network, which seems to facilitate the encounter, as the group acts as the promoter of the context for the meeting to take place. Young adults may find themselves acting under the pressure of their social network (Luz et al., 2022) and may incur more health risk from behaviors such as unprotected sex (Holman & Sillars, 2012). Flirting is one essential part of this script, constituting a very detailed sub-script. Because partners are mostly strangers and communication is mainly non-verbal, this step-by-step guide may represent a way to make explicit and reassure both individuals about the expected sexual intentions and interactions. Besides physical attraction, other motivations are considered central elements in this script, such as the lack of affective and/or sexual interactions, or looking for new experiences. A less-expected finding, but one that is in line with the evidence of transitions occurring between different types of relationships (Hadden et al., 2019), is that this script includes the possibility of repeated encounters when partners enjoyed being together. This feature may indicate that, despite a lower emotional involvement in this CSR when compared to *hookup* and, especially, *friends with benefits*, the *one-night stand* is neither as straightforward nor as totally detached as its definition may suggest, endorsing the idea that being good sex partners may (emotionally) bond CSR partners (Rodrigue et al., 2018). This finding puts forward the possibility of a sexual encounter that more frequently associated with risky health and sexual behaviors (e.g., alcohol and substance use, unprotected sex with strangers) may also be a potential basis for sexual activities and expression that lead to enjoyment and (sexual) pleasure due to its free and spontaneous character – a finding that endorses the need to change the discourse around sexuality to include the beneficial aspects of sexuality (Ford et al., 2021; Gianotten et al., 2021).

Rules are part of the *friends with benefits* and *hookup* scripts and are mostly focused on ensuring that CSR partners have no obligations nor are (romantically) committed to each other, and that the relationship remains in the intimate sphere. Given that one risk associated with CSRs is the development of romantic feelings (Luz et al., 2022), when there are repeated encounters, it may be important to both partners to make explicit that these relationships are meant to be uncommitted and entail neither personal responsibilities nor public awareness of them. Nevertheless, the development of romantic feelings by one of the partners is scripted as a frequent outcome in *friends with benefits*, probably due to the stronger and more profound emotional bond, which more often serves as a reason to terminate the relationship than to transition to a committed one (Machia et al., 2020).

Despite presenting a significant number of central actions, the *hookup* script seems to entail a certain level of ambiguity, not only regarding the sexual behaviors that may occur, but also in relation to a number of features, such as the place where the encounters occur, the partner type, and the emotional involvement that may (or may not) lead to sexual involvement. These script elements seem to increase the variability of subsequent steps in each moment of this CSR.

Since scripts guide individuals’ expectations about what will or will not occur during the social and sexual interaction through a number of logical if-then statements (Bargh, 1996; Schank & Abelson, 1977), and since individuals’ motivations concerning casual sex may influence their inferences about the other’s sexual intent (Lenton & Bryan, 2005), potential CSR partners subject each other to ongoing evaluation in order to make accurate judgments about each other’s (sexual) intentions, in order not to develop wrong expectations, nor to initiate undesirable or unexpected behaviors.

Sexual contact in the three scripts includes different sexual practices, which is in line with the literature that highlights significant levels of passion in different types of CSR (Rodrigue et al., 2018). However, the three scripts can be distinguished according to the pace of the development of the relationship toward sex and subjective experience of sex, likely related with the motivations each participant has in engaging in one of these relationships and the CSR partner. Additionally, the *hookup* script is distinctive in that kissing might not happen until there is some level of intimacy, and not only does sexual intercourse not occur in the first meeting, it may not happen at all. This finding is also aligned with the literature concerning CSRs where “only a minority of these encounters appear to involve oral sex and intercourse” (Bible, 2022, p. 1778).

### The condom in the scripts

We expected the use of condom would not be part of all CSRs scripts, being present only in the script for *one-night stand* and absent in CSRs with a higher partner familiarity. Our findings partially meet this expectation, as condoms were included in the *one-night stand* script, as expected, but also in the *friends with benefits* script, being absent in the *hookup* script. Based on these results, we may conclude that sexual risk behaviors are less expected when individuals are involved in CSRs (as opposed to committed relationships), although this statement has to be interpreted with caution as other factors may have influenced findings, such as social desirability or the consideration of one’s own experience rather than what usually happens in each type of relationship.

The fact that condoms are not part of the *hookup* script reflects the variability of typical behaviors in this CSR, as well as its equivocation in what concerns sexual practices and the occurrence of sexual intercourse, making condom use less frequent than in the other scripts.

In the *one-night stand*, condom use is an expected behavior, but the reference to being used with people who do not know each other is somewhat ambiguous; it may mean that in the *one-night stand* it is always used because they are unknown partners, or it may be used only if they do not know each other. It therefore remains unclear whether it is used and whether this action is indeed part of the *one-night stand* script. Considering that the spontaneous nature of sexual interactions with little communication between some CSR partners and the use of alcohol may give rise to unsafe sex behaviors (Holman & Sillars, 2012; Skakoon-Sparling et al., 2016), we hypothesize that this result may be, in part, an effect of social desirability (Agnew & Loving, 1998), despite the inclusion of explicit study instructions emphasizing participants’ anonymity and instructing that responses should reflect what usually happens in a CSR rather than in personal experiences.

Also, contrary to what we expected from the evidence showing that condom use becomes less common as casual relationships continue (Lima et al., 2018), and that friendship contributes to partners forgoing condom use (e.g., Vanderdrift et al., 2012), using a condom is reported as quite typical in the *friends with benefits* script. This notwithstanding, it is also typical that partners talk about using (or not using) a condom, which seems to leave open the possibility for not using it. Once again, this finding includes some ambiguity in the way it is reported, so the presence of condoms in the *one-night stand* and *friends with benefits* scripts merits further investigation.

### Strengths and limitations

We set out from the possibility of different scripts guiding casual sexual interactions. To our knowledge, this is the first study that, using a mixed-methods approach, simultaneously explores and compares the sexual scripts of the main types of CSRs whose labels and distinctive features were previously clarified and validated (Alvarez, Pereira, et al., 2021). Participants were emerging adults, not only college students, which may confer a broader understanding of the casual sexual scripts among this population. In addition, although it was not equal, the percentages of men and women were close, and around a half of the sample had already been involved in at least one CSR.

There are also some limitations that must be considered. We did not conduct a gender-driven analysis, which could have provided more information concerning some level of gendering of the casual sexual scripts, such as the existence of gender norms. And, as stated before, there may have been a social desirability bias in some participants’ contributions, mainly regarding the actions that are highly recommended, such as condom use in casual sex, arising from the number of explanatory conditions for its use.

## Conclusions

This study adds to prior work in deepening knowledge about the multiple sexual scripts that guide expectations and behaviors during casual sex encounters. The structure of the *one-night stand*, *friends with benefits*, and *hookup* scripts is comprehensively described, putting in evidence the complexity and richness of each script, and pointing to consensual cultural knowledge about the actions and events that are typically present in each type of CSR. The three casual sexual scripts are hence constituted by strong script elements, being also qualitatively different as they only share a few script elements. This clearly adds to the evidence pointing to the diversity of CSRs. However, given the dynamic and porous boundaries between CSRs (Epstein et al., 2009; Luz et al., 2022), evidence of different sexual scripts does not mean that individuals know from the beginning of the encounter which type of relationship they are getting involved in, namely when it comes to the *one-night stand* and *hookup*. Condom use is present in the scripts where sexual intercourse is equally expected, but due to some level of uncertainty concerning its effective use, further studies should be conducted to clarify its presence in each script.

Our findings on multiple casual sex scripts may inform sex education programs in extending perspectives on sexuality beyond risk prevention and clarifying the positive and pleasurable aspects of sexuality that are present in different casual sexual relationships, as well as in developing communication and decision-making skills concerning sexual choices, using specific knowledge of the CSR scripts to promote sexual health and wellbeing.

## Ethical approval

All procedures performed in studies involving human participants were in accordance with the ethical standards of the institutional and/or national research committee and with the 1964 Helsinki declaration and its later amendments or comparable ethical standards.

## Informed consent

Informed consent was obtained from all individual participants included in the study.

Data are available upon request from the first author.

## Supplementary Material

Supplemental Material

## Appendix

### Friends with benefits definition

Relationship between two people who already have a previous friendship, with trust between the parties, but without romantic feelings involved.

### Hooking up definition

Spontaneous and less-planned encounter, which may occur between acquaintances or strangers and may happen more than once with the same person. Rules are not established, and oftentimes penetrative sex is not involved (only kissing, embracing, and caressing).

### One-night stand definition

A sexual encounter between strangers who will not see each other again, usually accompanied by the consumption of alcohol and/or drugs in night spaces, with attraction being the triggering factor of the relationship.

### Friends with benefits sexual script

Both feel friendship, respect, and trust for each other. There are also feelings of warmth and affection, and when there are signs of mutual interest, they message each other. The two of them start by talking and communicate well and often.

The encounter occurs at night and happens because there has been physical attraction for some time and both of them want to have sex without being in a relationship. In the beginning there is kissing, necking touching, embracing, and caressing, and they have sexual intercourse. There is sexual compatibility because they already know each other. There is also mutuality and sexual ease, and there is room for exploration of sexuality. There is a willingness to arrange sexual encounters and to talk about whether or not to use a condom. If yes, they check if there is a condom, and they use it.

After having sex they talk, they find out if the sex was good, they talk about ordinary, everyday things, and they relax together. They arrange their sexual encounters by text message. Few or no friends know about the relationship, and in social situations they hide that they have a sexual relationship (for example, by avoiding physical proximity). No explanations have to be given to each other.

Usually, one partner develops romantic feelings. The relationship ends because one partner develops romantic feelings and the other does not. When one has a romantic interest, the one who doesn’t become distant in order to end the sexual encounters. Sexual encounters also end because one person is interested in someone else. To end sexual encounters the people are together less often. They text less often or stop texting.

### One-night stand sexual script

It happens on a night out with groups of friends with the intention of socializing with people who do not know each other. In places with many people the physical proximity is conducive to physical contact that occurs apparently by accident.

In a disco, they drink alcohol, smoke, and dance in groups of friends. At some point, people from one group, usually of one gender, mingle with people from another group, usually of another gender. One tries to seduce someone who is at the same level of physical appearance and shows interest. They show interest by holding their gaze. They might first try a quick glance to see if the other is looking at them. They exchange a gaze and address the person.

The first contact can be very natural and spontaneous – they take a selfie together – and from there they strike up a conversation, or one gets close to the other and starts chatting. They offer a drink or invite the other to go have a drink. They dance with each other. They whisper in the other’s ear, compliment them, say a funny line, or give them something to show interest.

It includes seduction, connecting, excitement, flirting, and kissing. Communication is nonverbal, with exchanges of glances and bodily attraction. They hint at sexual interest with provocative conversation, flirting, and seduction. They demonstrate and evaluate the other person’s interest indirectly through physical contact. There is physical proximity to demonstrate attraction or interest. They assess the other person’s availability and interest. They ask if the person wants to get out of there and go somewhere else.

The encounter happens because there is physical attraction, sexual tension, and because there is emotional and sexual need and loneliness. They are looking for new sensations and experiences. It also happens because they are single or because they haven’t been in a relationship for a long time.

The goal is to have sex without commitment. There is no emotional involvement: they arrive, they feel like it, they do it, and they leave. There are no obligations or “demands”. They have sexual intercourse. The sex is fast and intense.

They always carry a condom with them. They carry a condom with them when they anticipate or plan to have sex. They use a condom with people they don’t know. They always use a condom.

They exchange social networks. They exchange cell phone numbers. There are more encounters if they both want.

### Hookup sexual script

They meet in a social context or on social networks. They initiate contact and show interest in dating apps and social networks. They talk regularly (exchange messages) in chat and social networks and use profiles and posts to learn more about each other. They press “like” on photos, return likes, or respond to a story. They text each other to ask each other out: for coffee, to each other’s houses, or to go for a drive. They talk to each other to start feeling at ease. They talk about topics or interests in common, tell light, funny stories, laugh together, and show interest with their gaze. It can also happen on a night out, at a party, festival or concert. They feel physical attraction, but not passion.

They do not kiss or hold hands on the street, and they do not act like partners in front of most people. This relationship does not imply commitment and can end at any time.

When there is already some intimacy, they start by kissing. On a first date they may only kiss, and then touch and feel each other. After a date where they only kiss, they have sex.

To end the hookup, people become less interested, slowly stop answering messages or take longer to answer them. They may also say that they don’t want more and end the relation.
