# Supplementary material for: Multiple Casual Sex Scripts: Shared Beliefs about Behavior among Portuguese Emerging Adults
Source: Int J Sex Health. 2023 Feb 2;35(1):105–22. doi: 10.1080/19317611.2023.2172512 (PMC10903638; doi:10.1080/19317611.2023.2172512)
Supplement: Supplemental Material [file WIJS_A_2172512_SM3751.docx]

Supplementary File

Script actions for *One-night stand*, *Friends with benefits* and *Hook-Up*, Study 1 (focus group interviews and list completion) and Study 2 (consensual script actions; *60-64.99%, **65-69.99%, ***≥70%)

| Moment | Category | Task | Script actions | | |
| --- | --- | --- | --- | --- | --- |
|  |  |  | One-night stand | Friends with benefits | Hook-Up |
| CONTEXT | Time and moment | Focus groups | It happens on a night out.**  They drink alcohol.*  They’re drunk.  They’re at a club.  Apps speed up these relationships. | The encounter happens at night.*  The encounter happens at a club.  The encounter happens at home. | It happens as much during the day as at night.  They go out for a drink or a coffee.*^1^  They're at a café.  They engage in activities where they can talk. |
|  |  | Free-recall | They are under the influence of drugs.  They are needy or lonely.  They dance together. | The encounter happens at places such as in the car, at a party, at a café. | It can happen on a night out.***  It can happen at a party.***  It can happen at a festival.*** |
|  | Emotional involvement | Focus groups | The aim is to have sex without commitment.***  There is no emotional involvement.** | There are no romantic feelings.  Both just want to have sex.  Both feel respect and trust for each other.** | They feel physical attraction, but not passion.***  They care about what the other person is feeling.  There is more intellectual attraction than (physical) attraction. |
|  |  | Free-recall | There is no obligations or demands.**  No exchanging contacts or seeing each other again.  The goal is sexual satisfaction only.  It includes seduction, hooking up, arousal, flirting and kissing.* | In these interactions there is a feeling of friendship.*  In these interactions there are no feelings of exclusivity.  In these interactions there are feelings of care and affection.*  At first there is fear and guilt. | There is more physical than intellectual attraction. |
|  | Partner | Focus groups | One tries to seduce someone who is on the same level in terms of physical appearance.***  It makes it easier to be predisposed to interaction if the other person is already known on social networks.  One avoids a relationship with someone who publishes a lot of pictures on social networks.  One’s friends influence the choice. |  | They meet through a mutual friend.  They meet in a social context.**  They look for information about the other person (e.g. on social networks). |
|  |  | Free-recall | One tries to seduce someone who shows interest.***  The other person has to be someone who is capable of not committing.  The other person is sober. |  | They get to know each other online or in social networks.  They are friends, colleagues, or long-time acquaintances. |
| APPROACH | Tecnhology use | Focus groups | You check their social media profile to see how they might be approached at a party or club.  They use the apps to see who matches and they set up the meeting.  It helps to know what to expect from the person from the information they share on social networks.  They get messages and pictures on social networks inviting them to a date.  They receive and send nudes.  At a club, they can tell that the other person is interested when they reciprocate the likes on social media. | The people start by following each other on social networks.  When there are signs of mutual interest, they send a message.** | They meet on social media.**  They press “like” on each other's photos.**  They reply to a story.**  They send message asking each other out.**  They infer what kind of relationship can be had by what is posted on Instagram, Twitter, Facebook. |
|  |  | Free-recall | There is online chat beforehand to get to know the person better.  Technology is not used so that there are no connections to our social networks.  There are digital preliminaries. | At first they exchange likes on photos and social network posts, or react to stories.  They use technology to exchange intimate messages, photos, and videos.  Friends with benefits rely on dating apps. | They talk regularly (exchange messages) on chat and social networks.*  Use profiles and posts on social networks to find out more about each other.*  They initiate contact and show interest on dating apps and social networks.*  They send intimate photos and share sexual fantasies. |
|  | Social Network | Focus groups | Going out with groups of friends with the intention of socializing with people they don't know.*  Going out to a nightclub in groups of the same kind.  At a club, drinking, smoking and dancing in groups of friends.***  At a club, friends serve as go-betweens and create a bond between two people.  At a club, a friend notices that someone is interested.  At a club, at some point, people from groups of one gender mingle with groups of the other gender.*  At a club, each one leaves his or her group of friends and goes for a drink with the other person.  At a club, each one leaves their group of friends and goes dancing with the other person.  At a club, there are have friends who are “on the alert” to turn to if something goes wrong. |  |  |
|  |  | Free-recall |  |  |  |
|  | Planification | Focus groups | The meeting can be planned through messaging.  The first contact can be very natural and spontaneous (taking a selfie together) and conversation begins from there.*  The encounter may be planned because there is previous physical attraction.  The person is mentally prepared for sex to happen that night.  The encounter can happen without there being prior availability. | The sexual encounter happens unexpectedly. | They invite the other for coffee.* |
|  |  | Free-recall |  | The sexual encounter is planned by the partners.  The initiation of the sexual encounter is discussed. | They invite each other over to their place.*  They invite each other for a drive.*  They arrange to go to the movies.  It isn't planned, it just happens. |
|  | Flirt | Focus groups actions | Glancing (looking unseen to see if the other person is looking).***  Exchanging looks.***  Showing interest by holding the gaze.***  Facing the person.***  Starting conversation.***  In crowded places, physical proximity promotes seemingly accidental physical contact.***  Offering a drink.*  Inviting the other person for a drink.* |  |  |
|  |  | Free-recall | Dancing together.*  Behaving in ways that attract attention.  One whispers in the ear and compliments them. ** |  |  |
|  | Verbal communication | Focus groups actions | One approaches the person by making conversation.***  Asking directly if the person wants to have a sexual experience with oneself.  Never being direct about what is wanted.  No conversation, only action.  If they start by talking, the conversation happens, if they don't start by talking, then they don't talk and move on to the next step.  They ask if the other person wants to get out of there and go somewhere else.* | Starting to converse.*** | They share more intimate experiences.  They talk to each other to start feeling at ease (with a cup of coffee, for example).** |
|  |  | Free-recall | Sexual interest is indicated with provocative conversation, flirting, and seduction.***  The communication is nonverbal, with glances exchanged and bodily attraction.** | They communicate well and often.*  They are clear about what they both want from the relationship.  There is little communication. | They talk about subjects or interests they have in common.***  They tell light, funny stories.***  They laugh together and show interest with their gaze.*** |
|  | Motivation | Focus groups actions | It happens because there is physical attraction.***  There is sexual tension.*** | It happens because there has been physical attraction for some time.** |  |
|  |  | Free-recall | It happens because there is emotional need and loneliness.*  It happens because there is sexual desire.***  New sensations and experiences are sought.**  The encounter happens because one is single.*** | Both want to have sex without being in a relationship.**  It happens because a physical attraction occurs at a moment’s notice or unexpectedly.  It happens because they have a special connection, but no deeper feelings.  It happens because they have a good relationship and mutual trust.  It happens because they want to have more sexual experience. |  |
|  | Defining rules | Focus groups actions |  | There is a willingness to arrange sexual encounters.*  It is clarified that there will be no romantic involvement.  There are explicit agreements between the two people about the rules, boundaries, and expectations for the relationship (e.g., no infatuation).  The boundaries of the relationship are established at the start (for example, that it will be a casual relationship).  There are implicit rules (e.g. the sexual encounter only happens when it is arranged, one should avoid frequent contact with the other).  In social situations, they disguise that they have a sexual relationship (for example, by avoiding physical proximity).** | They make their intentions explicit before moving on to a sexual relationship.  They go out to dinner with friends.  They do not kiss or hold hands on the street.***  They know that after going out with their friends, they will have sex. |
|  |  | Free-recall |  | Few or no friends know about the relationship.*  No explanations have to be given between the two of them.**  They talk about the (non)exclusivity of the relationship. | They do not act like partners in front of most people.***  This relationship does not imply commitment and can be ended at any time.*** |
| SEXUAL EXPERIENCE AND USE OF CONDOM | First sexual contact | Focus groups actions | Demonstrate and gauge the other person’s interest indirectly through physical contact.**  Invite them out for a drink and kiss.  They get together on the dance floor and start kissing.  They start kissing without conversation. | They kiss on the lips, cuddle, kiss with tongue.*** | When there is already some intimacy they start by kissing.***  On a first date (e.g. in the evening) they may only kiss a few times.*** |
|  |  | Free-recall | There is physical proximity to show attraction or interest.***  They evaluate each other's availability and interest.*** | They have sex.***^1^  There is caressing, hugging, cuddling..***  They hold hands in public. | After a few kisses they touch and feel each other.*** |
|  | Sex | Focus groups actions | They have sex.*  They get involved in the club and go home or to their car or to a friend's house (to have sex).  The sexual experiences are typical scenes. | They have sex.***  There is sexual compatibility because the people already know each other.* | After a date in which they only kiss, they have sexual intercourse.* |
|  |  | Free-recall | The sexual intercourse is quick and intense.*  They have sex in a public place. | There is room for exploration of sexuality.***  There is affinity and sexual ease.** | Sexual intercourse occurs in a variety of places, including public places such as a parking lot. |
|  | Use of condom and communication | Focus groups actions | One uses condoms with people one doesn't know.***  They always use a condom.***  They always carry a condom with them.*  Not having a condom does not stop them from having sex.  They can use a condom on the same night in one relationship and not in another.  When there is no prior availability for sexual intercourse and it does occur, they might not use a condom.  They don't use a condom when they think they won't get infected. | They check if there is a condom.***  People are not comfortable talking about condoms.  Condoms are used.***  It is not necessary to use a condom because they know and trust the person, even when the friendship is not longstanding (for example, they know the person from college). |  |
|  |  | Free-recall | One brings a condom when one anticipates or plans to have sex.*** | There’s willingness to talk about whether they are going to use a condom or not.*** |  |
| AFTER SEX | Communication | Focus groups actions | The next day it is not necessary to clarify anything. | After having sex, they talk.**  They talk about what they expect from the relationship so that there are no false expectations.  They clarify when romantic feelings arise so that they understand if they are mutual, and no hurt feelings arise. |  |
|  |  | Free-recall | The next day you clarify what happened.  They do not talk the next day.  They make small talk or polite conversation the next day. | After having sex, they check if the sex was good.*  They talk about ordinary, everyday things.*  After having sex, they relax together.* |  |
|  | Technology use | Focus groups | Social networks are exchanged.* | They arrange sexual encounters by text message.***  They text less frequently to end the relation.* |  |
|  |  | Free-recall | Cell phone numbers are exchanged.* | They phone. |  |
|  | Outcome with partner | Focus groups actions |  | There may be other relationships at the same time, but they are more casual.  The relationship may be monogamous.  One partner usually develops romantic feelings.** |  |
|  |  | Free-recall |  | The relationship is not monogamous. |  |
|  | Continuity and duration | Focus groups actions | The one-night stand turns into a relationship.  There are new encounters if both want.*  There's no contact again even if they liked it.  There is no contact after the encounter. | It can last a long time.  It may happen only once.  The relationship can become more serious. | They have a list of people with whom they have a hook-up type relationship.  After having sex, the people don't get back in touch. |
|  |  | Free-recall |  | The relationship occurs infrequently. | They continue to meet from time to time to have sex.  After the sex happens a friendship comes to be created.  It evolves into something more (e.g. boyfriend/girlfriend, public but unlabeled relationship).  They don't get back together sexually. |
|  | Ending | Focus groups actions |  | The relationship ends because one of the partners enters into a committed relationship.  It is common for the encounters to end because one of them develops romantic feelings for the other.*  To end the sexual encounters, the people drift apart.*  To end the sexual encounters, the people stop sending messages.*  To end the sexual encounters the people get together less often.**  To end the sexual encounters, the people openly say they don't want to continue.  When one person has a romantic interest, the one who doesn't have it distances themselves to end the relationship.*  When the sexual relationship ends, the people remain friends.  When the sexual relationship ends, the friendship ends. | To end the relationship, they gradually stop responding (to messages).*  To end the relationship, they take longer to respond (to messages).* |
|  |  | Free-recall |  | The relationship ends because there ceases to be attraction.  The relationship ends because one person is interested in someone else.* | They say they don't want it anymore and end it.*  To end the hookup they show less interest.***  To end it, they ghost the other person. |
| Total number of actions per script | | | 44 actions ≥60% | 35 actions ≥60% | 30 actions ≥60% |
| ^1^the content of the script action is repeated | | | | | |
